# Cultivating resilience and hope: A qualitative study of a pilot program using patient navigators to assist men who have sex with men with retention in the HIV care continuum in Uganda

**DOI:** 10.1371/journal.pgph.0001475

**Published:** 2023-01-19

**Authors:** Markus Larsson, Arielle N’Diaye, Richard Lusimbo, Anette Agardh

**Affiliations:** 1 Division of Social Medicine and Global Health, Department of Clinical Sciences, Lund University, Malmö, Sweden; 2 Uganda Key Population Consortium, Kampala, Uganda; University of Bremen: Universitat Bremen, GERMANY

## Abstract

In Uganda, due to the criminalization of same-sex sexual practices, men who have sex with men (MSM) experience barriers to accessing HIV care. To retain patients within the HIV Care Continuum, some health interventions have used patient navigators as an ancillary support service. To understand the potential care benefits of using patient navigators for marginalized populations experiencing challenges to HIV care and treatment access in a Ugandan context, this qualitative study explored the experiences of newly diagnosed MSM using patient navigators for ARV retention in care in Kampala. Additionally, to gain insight into the feasibility of patient navigator interventions, this study also aimed to understand the perspectives and experiences of patient navigators working with HIV positive MSM. Individual in-depth, semi structured interviews were conducted with 24 HIV positive MSM and four patient navigators that were part of a patient navigator pilot program from January 2019 –December 2020. Analysis was done using manifest and latent qualitative content analysis. Results showed that HIV positive MSM in Uganda experienced a variety of social, emotional, and financial challenges that placed them at risk for dropping off the HIV Care Continuum. Patient navigators provided HIV positive MSM with the skills, support, and resources necessary to overcome these challenges. Based on study results, we conclude that within the patient navigator pilot program, patient navigators improved MSM participants’ quality of life by helping them to achieve the HIV Care Continuum stages: diagnosis, linked to care, receiving HIV treatment, and retention in care. Study results suggest future research is needed on the psychosocial support needs of patient navigators, how the support needs of MSM change throughout their lifetime on the HIV Care Continuum, and how potential benefits of patient navigators may differ in rural Ugandan contexts.

## Introduction

The HIV Care Continuum is a model that describes HIV treatment and management in five stages: *Diagnosis*, *Linked to Care*, *Receiving Treatment*, *Retainment in Care*, *and Achieving Viral Suppression* [1–3]. Some studies have used it as a tool to investigate opportunities to better operationalize HIV specific health and social services [2, 3]. To retain patients within the HIV Care Continuum, some health systems have used patient navigators (PNs) as an ancillary support service [4, 5].

PNs are individuals tasked with linking patients to health and social services, minimizing barriers to care, ensuring patient adherence to treatment plans, increasing patient health literacy, and reducing patient fears [6–10]. Additionally, PNs provide a sense of continuity to patients as they navigate health and social service systems and have been effectively used to manage chronic conditions such as cancer, diabetes, cardiovascular disease, and HIV [7–12]. Existing scientific literature on PNs has identified examining patient experiences and intervention feasibility as areas for further study [11]. In contexts where same-sex sexual behavior is highly stigmatized, PNs have been used to both provide information about HIV and assist with gaining the skills needed for living with HIV. Set in Guatemala, Nigeria, and Kenya, these interventions successfully motivated patients to remain engaged in care, linked newly diagnosed patients to care, addressed antiretroviral therapy (ARV) adherence barriers, and provided emotional support [4, 5, 13].

In 2020, it was estimated that 24,100 men who have sex with men (MSM) were living in Uganda, with an HIV prevalence rate of 13.2% [14]. Among MSM in Uganda, it has been estimated that 54% were aware of their HIV status, and 66% of MSM diagnosed with HIV were receiving ARVs [14]. However, due to the criminalization and stigmatization of same-sex sexual behavior, official demographic information on this population is scarce [15]. Moreover, due to the stigmatization and criminalization of same-sex sexual practices, HIV positive MSM in Uganda face many barriers that hinder their access to ARVs [16–18]. These barriers include stigma and homophobia, difficulty affording care, and low numbers of health workers sensitized to meet MSM specific health needs [17–21]. In healthcare settings, MSM often feel uncomfortable discussing their sexual practices with health workers and fear being denied treatment if health workers learn of their sexual history [19, 20]. For some MSM, internalized stigma and health worker discrimination lead them to completely forgo accessing health services [20]. These barriers together with high levels of geographic mobility among MSM have made necessities such as lubricants, ARVs, and treatments for sexually transmitted infections (STIs) difficult to access and retention in care challenging [19]. To better understand the potential care benefits of using PNs for marginalized populations experiencing challenges to HIV care and treatment access in a Ugandan context, this study aimed to explore the experiences of newly diagnosed MSM using PNs for ARV retention in care in Kampala. Additionally, to gain insight into the feasibility of PN interventions, this study also aimed to understand the perspectives and experiences of PNs working with HIV positive MSM.

## Materials and methods

### Study setting

This study was part of a PN pilot program that took place in Kampala, Uganda, from January 2019 to December 2020. The PN pilot program had the overall goals of 1) helping MSM newly diagnosed with HIV to make informed decisions about their healthcare seeking practices, and 2) aiding newly diagnosed MSM to understand how to navigate the healthcare system to access medication and treatment. Because this research study was part of the PN pilot program, everyone involved within the pilot was also part of the research study described in this article. A summary of the goals is presented in Table 1.

**Table 1:** Summary of research study aims and patient navigator pilot program goals.

Research study aims	Patient navigator pilot program goals
To explore the experiences of newly diagnosed MSM using PNs for ARV retention in care in Kampala	Help MSM newly diagnosed with HIV make informed decisions about their healthcare seeking practices
To understand the perspectives and experiences of PNs working with HIV positive MSM	Aid newly diagnosed MSM to understand how to navigate health system to access medication and treatment

### Study sample

PNs and MSM participants were recruited through purposive sampling for the PN pilot program and the study that took place within it. Eligible MSM participants were MSM over 18 years of age and newly diagnosed with HIV (one-month post diagnosis or less). Eligible PN participants were MSM over 18 years of age. For recruitment, the research team contacted an LGBTQ (Lesbian, Gay, Bisexual, Transgender, and Queer) umbrella organization (NGO) in Kampala that assisted with PN and MSM participant recruitment for both the PN pilot program and the study. In total, four PN participants were recruited, all engaged in different organizations working with LGBTQ populations. For their participation in the pilot program, PN participants were compensated with a stipend and received training by a medical professional associated with the research study. The training was conducted using existing WHO and UNAIDS protocols for explaining HIV and AIDS, treatment, stigma, and discrimination. This same organization then sourced potential MSM participants through their networks. Once a potential MSM participant fitting the selection criteria was identified, the organization informed them about the pilot program and the research study. In total 24 MSM participants were recruited.

Once data collection for the research study began, consent was obtained from all participants prior to beginning each interview. This recruitment protocol for both MSM and PN participants was considered appropriate given the sensitivity surrounding LGBTQ individuals in Uganda. Recruitment finished when it was agreed upon among the researchers that sufficient information power had been reached, according to the concept suggested by Malterud et al. [22].

### Data collection

Data was collected using individual in-depth interviews with two semi-structured interview guides (i.e., PN and MSM participants, respectively). The PN participant interview guide contained four parts: *their motivation and ideals; their perceptions of pilot participants; their relationship with pilot participants; and their experiences within the patient navigator pilot program*. The MSM participant interview guide contained three parts: *information about their experience living with HIV; their individual relationship with their patient navigator; and their experience within the patient navigator pilot program*. Further detail about these interview guides can be found in S1 and S2 Texts. Because the study aim strived to capture MSM participant experiences as they moved through different stages within the HIV Care Continuum, both MSM and PN participant interviews were conducted at two points in time (June 2019 and November 2019). These two points in time were chosen because it gave MSM participants six months to move to other stages within the HIV Care Continuum. The same interview guide was used on both occasions. Participation in two interviews was, however, optional. Interviews were conducted in person and in English with a Luganda speaking translator present. A total of eight interviews used an interpreter. In these interviews the interpreter was used to clarify questions posed by the interviewer in English that respondents did not fully understand. The first author (ML) conducted the interviews in June and November 2019, and the last author (AA) conducted the interviews in November 2019. Interviews were audio recorded and transcribed verbatim. The text sections in Luganda were translated by a member of the research team, fluent in both Luganda and English. The option to conduct the interview without an audio recorder was provided, but all respondents agreed to be recorded. Interviews occurred in a secure location within a private room. To protect respondent identities, no identifying data was collected during interviews. Additionally, all transcripts were anonymized prior to analysis and only members of the research team had access to audio recordings and interview transcripts.

### Data analysis

Transcripts were analyzed using manifest and latent qualitative content analysis with an inductive approach [23, 24]. Study data was analyzed from the perspective of pilot participants moving through the HIV Care Continuum. As a result, both June and November interviews were analyzed together. For the coding process, individual interviews were the units of analysis, and meaning units in the form of sentences and paragraphs were condensed and labeled with codes. Codes were grouped into subcategories and combined to form categories. The content of these categories was examined to identify emerging subthemes and themes. Analysis was initially conducted by one member of the research team (AN). Following this, the analytical model was examined by the other co-authors (AA, ML, RL) to obtain feedback and consensus on study findings.

### Researcher characteristics and reflexivity

Among the co-authors, two are Swedish and based in Sweden (AA, ML), one is American and based in the United States (AN), and one is Ugandan and based in Uganda (RL). All co-authors have previous knowledge and experience of working in the areas of HIV and key populations within a variety of Sub-Saharan African contexts.

This study was conducted in accordance with the Standards for Reporting Qualitative Research (SRQR) checklist [25]. As a result, steps were taken by the research team to reflexively examine how their positionality could potentially influence study outcomes. Because of his experience as a Ugandan working with key populations in Kampala, RL brought an insider perspective to this study’s design, analysis, and reporting processes. Since interviews were conducted by two non-Ugandan co-authors (AA, ML), having a Luganda speaking interpreter ensured that socio-linguistic nuances were mutually understood by both interviewers and respondents. Additionally, previous research experiences on the topics of HIV and key populations in Uganda had familiarized these two co-authors with the socio-cultural dynamics experienced by our study population. While conducting the initial analysis AN used a reflexive journal to describe analytical decisions, in addition to thoughts and questions that arose. The contents of this journal were discussed among the co-authors while examining the analytical model to mitigate the possibility of bias resulting from being an outsider to Ugandan socio-cultural dynamics. This reflexive process was also used when drafting and revising this manuscript.

### Ethical considerations

This study received approval from St. Francis Hospital Nsyamba REC: UG-REC-020 in Kampala, Uganda, and was performed in accordance with the principles of the Declaration of Helsinki [26]. Because this study involved a vulnerable research population, PN and MSM participants received thorough information about the voluntary and anonymous nature of their involvement. A representative from the collaborating partner organization that works with key populations met with prospective study respondents to explain the study purpose, potential risks, benefits, and their right to withdraw from the study at any time during the interview process or afterwards. None of the participants recruited chose to withdraw from this study. Information about the study and the terms of participation was also provided by the interviewer prior to beginning each interview. Respondents were also given the opportunity to ask questions on both occasions. In the event negative emotions arose during an interview, a member of the research team was available to connect all participants to a counsellor that would provide free of cost counselling. This information was also provided in the information letter.

## Results

39 interviews were conducted among 28 respondents (4 PN and 24 MSM participants), where the mean age among respondents was 29 years of age. 17 respondents (17 MSM participants) were interviewed once (either in June 2019 or in November 2019), and 11 respondents (4 PN and 7 MSM participants) were interviewed both in June 2019 and November 2019. In total, respondents ranged between 20–42 years of age. All respondents (both PN and MSM participants) self-identified as MSM; however some specifically identified as gay or bisexual. Respondent demographics are further described in Tables 2 and 3.

**Table 2:** MSM participant demographics.

Variable	N
**Sexual Orientation**	
Gay	18
Bisexual	1
Unknown	5
**Employment Status**	
Employed	4
Unemployed	15
Self-employed	3
Unknown	2

N = 24 MSM participants

**Table 3:** PN participant demographics.

Variable	N
**Sexual Orientation**	
Gay	2
Bisexual	1
Unknown	1
**Employment Status**	
Employed	4
Unemployed	0
Self-employed	0
Unknown	0

N = 4 PN participants

### Overarching theme: Cultivating resilience and hope against enduring trauma and stress

**Fig 1:**
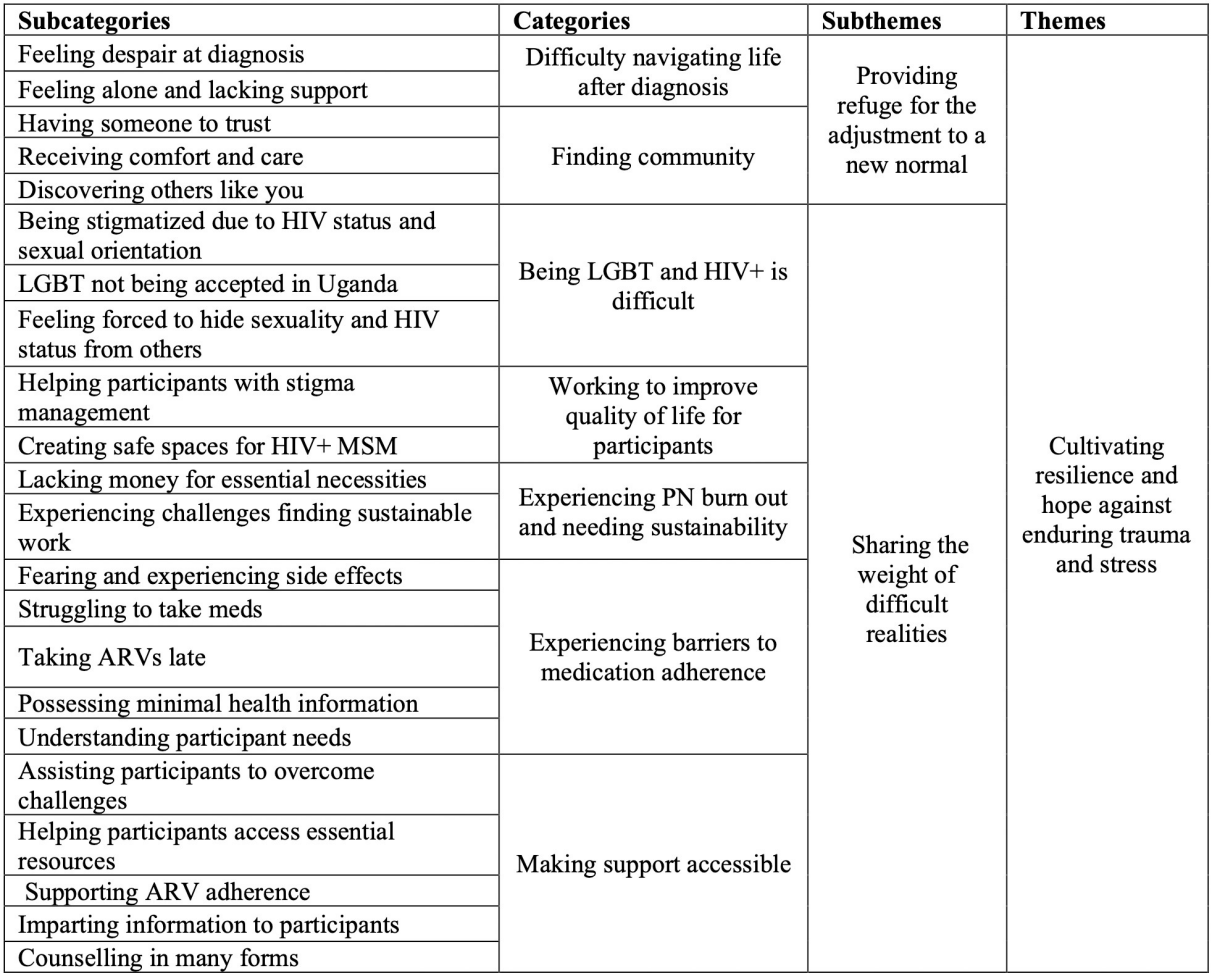
Analytical model of subcategories, categories, subthemes, and themes.

Data analysis resulted in one overarching theme, two sub-themes, seven categories, and 22 subcategories. The overarching theme *Cultivating resilience and hope against enduring trauma and stress* captures how the PN participants assisted MSM participants with developing their capacity to be resilient and remain hopeful in a social context where they are at risk for experiencing traumatic encounters and stress during their journey through the HIV Care Continuum. This theme illustrates how it is through strengthening these capacities—in tandem with the social and resource support network that the PN pilot program provides—that MSM participants are aided in remaining within the HIV Care Continuum despite the potential life challenges they may face. The overarching theme is supported by two subthemes: *Providing refuge for the adjustment to a new normal* and *Sharing the weight of difficult realities*. These subthemes are supported by seven categories and 22 subcategories that are described in the analytical model shown in Fig 1.

#### Subtheme 1: Providing refuge for the adjustment to a new normal

Post diagnosis, the PN pilot program provided MSM participants with a space in which to acclimate to life with HIV. Prior to enrolling in the program, learning to live with HIV was a difficult and lonely process for MSM participants. However, after meeting their respective PNs, MSM participants were able to find community both with their PN and with people who also identified as gay/bisexual and living with HIV. This subtheme represents both how MSM participants were given a dedicated safe space to process life post-diagnosis, and why they felt this space was needed.

*Difficulty navigating life after diagnosis*. According to MSM participants, prior to participating in the PN pilot program, navigating life post-diagnosis was a difficult and lonesome experience. For them, diagnosis was a moment of crisis where they experienced feelings of despair. These feelings could be described as a spectrum of hopelessness, shock, malaise, being overwhelmed, confusion, and suicidality. Some participants mentioned having difficulty accepting their status, and others spoke of having difficulty accepting how they acquired HIV. For many MSM participants, diagnosis felt like it was the beginning of the end of their lives. Additionally, post-diagnosis, MSM participants reported feeling alone and completely lacking support. They mentioned feeling abandoned by others following their diagnosis, experiencing stress about how to move forward with their lives, and needing someone to be there for them.

“So, when the result came back positive, I added two things: I don’t have any support from family and /…/ I am now HIV positive, so I felt overwhelmed. I almost committed suicide.”

(Participant 1)

MSM participants also disclosed feeling distressed whenever they thought about how they were going to continue living, as they now knew they were HIV positive. PN participants similarly stated that recently diagnosed MSM participants were especially fragile and mentioned trying to be there for them as they adjusted to life post-diagnosis.

“/…/ These new clients, they really need us more than any other person. Because the first day you start taking your treatment is the worst day of your life. Whereby the whole first month of starting the treatment is really a bad month. Reason being, they get dreams, bad dreams, [and get] sick for that first month. They have a lot of thoughts, they think a lot, they grow small. So, when no one is there with them they almost go mad. So, whenever I participate in testing and I find that someone has been diagnosed as HIV positive, I always get closer to them so that I can be a backbone for them.”

(PN 4)

*Finding community*. After joining the PN pilot program MSM participants experienced finding community. Through their PNs, MSM participants felt they had someone they could trust. Even though some MSM participants were initially shy and hesitant to open up to their PN, PN participants were eventually viewed by all MSM participants as someone they could call at any time. MSM participants described PN participants as someone with whom they could talk about all aspects of life. Among PN participants, knowing MSM participant needs, secrets, and maintaining confidentiality were viewed as core competencies of their role. MSM participants mentioned how PN participants were trusted because of their patience, discretion, and their ability to support MSM participants. It is important to note that this trust between PN and MSM participants was mutual, where PN participants often shared personal details such as their HIV status and sexual orientation with MSM participants. Overall, both MSM and PN participants reported having good relationships with one another.

“Because sometimes people say, ‘Ah for me, no, no, no, let me stay in the closet’. But if he finds that the PN is a bisexual who talks about his sexuality and HIV openly, he feels more comfortable and then that’s how you start a conversation and you end up bonding.”

(PN 4)

Furthermore, many MSM participants perceived their PN as taking care of them like a family member and subsequently viewed them as a close friend or family member. PN participants shared a similar perception.

“I would say my relationship with the [participants], is good, it’s superb, it’s excellent. Because the way I handle my [participants], it’s like I am handling a real family, it’s like I am handling my real family. So, I do not feel that there is a challenge between me and my [participants].”

(PN 3)

Moreover, MSM participants described the PN program as somewhere they could receive comfort and care. This occurred through activities such as receiving home visits from their PN, visiting their PN at their home, being given words of encouragement, receiving counselling, being told that they were not alone, and being told that having HIV was not the end of their life.

“When I see and realize that someone has a big problem, I invite him to come and live with me for a while and when [he] gets better [he] can leave and go back to [his] place at home.”

(PN 1)

Almost all PN and MSM participants identified providing guidance and counselling as a core responsibility of PNs. According to MSM participants, counselling and encouragement played a large role in their adjustment process: “*I felt really good*. *Because [my PN] counselled me and told me that you are not alone*, *many people are sick out there*” (Participant 2). During counselling, MSM participants asked questions about how to live with HIV and expressed when they were feeling low. They described these activities as helping them to have a positive outlook on life and feeling like someone cared for them. Furthermore, from participating in the PN program MSM participants expressed discovering others who are also HIV positive and MSM. They articulated how PN participants created a support network of other HIV positive MSM in-person through one-on-one introductions and support groups, and online through WhatsApp groups. Through this network, MSM participants were able to form meaningful relationships by sharing their experiences of being HIV positive and LGBT. MSM participants found this network to be a source of encouragement and enjoyed meeting individuals among whom they could speak freely.

“Sunday, we meet somewhere, we talk, because all of us have HIV so we are free to talk. When you meet other HIV positive people, we are free to talk.”

(Participant 3)

#### Subtheme 2: Sharing the weight of difficult realities

While participating in the PN program, MSM participants experienced a variety of challenges. These included difficulties stemming from their sexual practices and HIV status, lacking basic material necessities, experiencing barriers to ARV adherence, and having limited access to treatment and health information.

To help MSM participants stay within the HIV Care Continuum, PN participants assisted them with overcoming these challenges. PN participants aided MSM participants with challenges relating to their sexual practices and HIV status by improving their quality of life. PN participants accomplished this by assisting with stigma management and creating safe spaces for HIV positive MSM. PN participants helped MSM participants overcome the challenges of lacking basic material necessities and barriers to ARV adherence by, for example, connecting MSM participants with individuals essential to solving their challenges and making resources accessible to participants.

*Being LGBT and HIV positive is difficult*. Both MSM and PN participants described being LGBT and HIV positive as a difficult experience. MSM participants feared stigmatization and recalled experiences of being doubly stigmatized for being HIV positive and MSM. MSM participants disclosed experiencing discrimination and being mistreated by health workers within hospital settings. It was because of this that some participants did not feel comfortable discussing their sexual practices with health workers.

“Because I feel that if I tell a health worker something that is related to my sexuality, that I am gay, he instead might shout it out loud in front of anyone. That’s my worry. That’s why I just don’t mention anything about it.”

(Participant 2)

“For us, the MSM, sometimes we are scared of going to the hospital for treatment because we do get beaten and physically assaulted in some of them. Like the recent scenario of the doctor beating his client, I reached a time when I got scared because I thought that maybe if I go to visit the medical facilities, I will also be beaten. So, I live in fear.”

(Participant 4)

MSM participants mentioned anonymity and privacy as important to them. They feared being seen by others at health facilities because not only did they face discrimination from general Ugandan society for their sexual practices, but also from the LGBT community for their HIV status. Both MSM and PN participants stated that being LGBT was not accepted within Ugandan society. MSM participants described fearing rejection from their families and being forced from home because of their sexuality. They also described feeling endangered, experiences of being outed on social media, and experiences of violence in reaction to their sexuality.

*Interviewer*: So, in the LGBT community, these [MSM participants] are also afraid of other community members finding out about their status?

*PN 4*: Yes, and yes. For example, some people will tell you that ‘I am comfortable with [my PN] picking up my food support and medication.’ [name of a health facility] is a place where they mostly get their medication, and they are afraid of being there. They will say, ‘what if I enter [name of a health facility] and I bump into my boyfriend and friends? So, for me I prefer [my PN] picking up [the food support and medication] for me’.

This fear of familial and societal rejection led many MSM participants to keep their HIV status secret. MSM participants feared what others would think about their status and feared that disclosure would lead to inquiries about their sexuality.

“For example, I am HIV positive, and I am still living with my family. So, if I tell them that I am HIV positive, they will throw me out. They will ask me how I got it, and if they find out that I got it as a *kuchu* [Luganda word for queer], they will tell everyone in the community, and I will become a reject in the entire community around my family.”

(Participant 6)

*Working to improve quality of life for participants*. PN participants sought to improve MSM participants’ quality of life by assisting with stigma management and by creating safe spaces for MSM living with HIV. One way in which PN participants helped MSM participants navigate stigma management was by guiding them with HIV status disclosure. This was done by providing counseling about HIV disclosure to participants and their families. PN participants also encouraged MSM participants to utilize health services and disclose their sexual practices to health workers they trusted. Additionally, PN participants reduced experiences of stigma by providing sensitivity training to health workers. Many MSM participants described waiting in line for long periods of time for ARVs as eliciting feelings of internalized stigma and fears of being stigmatized by health workers. In response to this, after consulting the PN participants, an LGBT organization created a voucher system for MSM participants to collect their ARVs without having to physically say that they are MSM. With health workers, PN participants often encountered the challenge of needing to conduct sensitivity trainings every few months due to frequent staff transfers between health facilities.

“But some, you know you train, you know you train medical staff, and in about three or four months, they are changed, they are changed and taken to another medical facility. So, you find that there are new ones there that are not oriented. So, at times there is an issue of the attitudes of health workers. That’s why we devise mechanisms of making those [ARV collection] vouchers. So that even if the attitude is there, there is not too much /…/.”

(PN 3)

“We do an orientation of healthcare workers on stigma, discrimination, and attitude change. So, when we diagnose a client, we [send] him to a focal point at a facility so that there is ease in getting a refill. The client goes and presents a coupon, or any other document and gets served fast. We have made it easier in the facility so that there is no queuing.”

(PN 1)

Overall, both PN and MSM participants voiced that assisting with stigma management and increasing participant comfort with health workers was seen as a core responsibility of PN participants.

“With doctors, the way [my PN] has been communicating with me has made me more confident [in] seeking for health services. My relationship with the care providers has improved, it is now good.”

(Participant 6)

To create safe spaces for MSM living with HIV, PN participants brought MSM participants to MSM friendly health facilities. At these facilities MSM participants felt comfortable speaking with health workers about their sexuality and did not experience discrimination because of their sexual practices.

“/…/ he has once talked to me about these people from [a local] hospital. They are called [name of organization]. He told me that if you have a problem, you can go to them, and they can help you.”

(Participant 11)

According to MSM participants, many MSM friendly health facilities provided a variety of services and worked in partnership with LGBT friendly organizations.

*Experiencing PN burnout and needing sustainability*. For PN participants, the high level of financial and emotional demands from MSM participants was sometimes overwhelming. According to MSM participants, finding sustainable employment was challenging and the majority reported being unemployed or underemployed.

Many expressed difficulty affording things like food, housing, mobile phones, and transportation and reported participating in activities like asking others for money to support themselves or engaging in sex work. As described by one PN participant, “*/…/ these are people who are needy*, *who are malnourished*, *who cannot even afford to buy a kilogram of rice*” (PN 4). For some PN participants, these feelings were exacerbated by challenges they were experiencing in their personal lives.

Moreover, listening to MSM participants’ traumas and hardships occasionally caused PN participants to relive their own past traumatic experiences. Likewise, PN participants also mentioned how they felt upset when they were not able to help MSM participants overcome their challenges. To help cope, PN participants participated in counselling and debriefed with each other. But despite these resources, some PN participants wished they had more substantial psychosocial support to help cope with these challenges.

“The biggest challenge I face as a patient navigator is the fact that maybe I should have some kind of retreat or some kind of period where I am off my duties and this period can be used to provide me with more capacity building and support in relation to HIV positive people. /…/ I do get some little support /…/ but sometimes I can really get overwhelmed by some issues especially [when] I have to receive a lot of problems from all of my [participants]. I get a bit overwhelmed or drained.”

(PN 2)

“It really affects me. Psychologically, it affects you as people come to you with tough situations. This is also because I went through the same challenges before, so I understand what they are talking about and what they are going through. Imagine someone telling you about something but you cannot provide enough help to relieve what they are going through.”

(PN 1)

*Experiencing barriers to medication adherence*. Furthermore, MSM participants experienced a variety of barriers to medication adherence. For some, experiencing side effects created challenges with ARV adherence. Many MSM participants were fearful of taking medication without food and consequently chose not to take their ARVs until they were able to find food.

“One time, one meal per day. If I have lunch, it’s breakfast, it’s supper. At times I miss the medicine because when I eat, I take the medicine.”

(Participant 8)

To help with this, PN participants provided participants with food assistance.

“He gave us support about food, transport costs. Whenever we are coming to support group meetings, he pays for us; he bought for us food, sugar, beans, posho, rice, etc.”

(Participant 5)

It is important to note that some MSM participants had trouble adhering to ARVs because of a general struggle to take medication regularly. They found the concept of being on medication for the rest of their lives as difficult to accept and something that caused them to lose hope.

“At first, I did not want to start any medication /…/. I personally have problems with swallowing or taking medication in general. And I was seeing that I will forever be on medication, I feared that too.”

(Participant 9)

Additionally, some MSM participants struggled with ARV adherence because they did not feel ready to start medication post diagnosis, they experienced inadequate counselling on ARVs at the hospital, or they felt that counsellors at health facilities were unavailable for drop-in inquiries. To help with these challenges, PN participants counselled MSM participants about the importance of taking ARVs and gave them the opportunity to ask questions.

“When he calls me. He doesn’t talk so much on phone but when I go to his place, he gives me a lot of advice. For example, why I need to adhere to my medication, what I need to do to keep my health better and keep my viral load low.”

(Participant 6)

Moreover, MSM participants struggled to take their ARVs because of additional medications they were taking for other illnesses they had. Lastly, MSM participants mentioned having difficulty taking their ARVs on time and frequently taking their ARVs late. This was attributed to challenges such as not being able to find transportation to collect their medication and preferring to take their medication in secret because they did not want others to know they were taking medication. As one MSM participant stated, “*Yeah*, *I have to hide [my ARVs]*. *I cannot tell people*. *Because I normally take it in the night*, *no one can see me*” (Participant 5). To help participants overcome these challenges, PN participants assisted MSM participants with collecting their medication and performed follow-ups to encourage medication adherence.

“The problem I had was that I was personally interested in receiving the medication from my village. […] [My PN] told me it is difficult, that it will difficult for me to get better services, He also told me that I needed to go to a place where he could be able to follow up and make sure that I am adhering to the medication […]You know, I think he suspected that I was afraid and would not manage to follow up on my medication. So, if I had taken my medication from the other side in the village, I wouldn’t have managed to take it, I wouldn’t have adhered.”

(Participant 7)

Furthermore, both MSM and PN participants mentioned that prior to participating in the PN pilot program, most MSM participants possessed minimal information about sexual health and life with HIV. They reported believing misinformation about life with HIV and receiving insufficient information about ARVs once diagnosed.

“The problem was that [a MSM participant] used to listen to rumors and certain myths, theories, and misconceptions from other people about the medication. But me, I explained to him how I managed to go through that period.”

(PN 2)

To combat misinformation, PN participants provided MSM participants with accurate sexual health information. As a result of this, MSM participants expressed having increased their knowledge about HIV and how to live with it. It is noteworthy that this information did not stay with MSM participants. Instead, MSM participants described sharing these lessons with others in their social networks.

*Making support accessible*. According to both PN and MSM participants, a key aspect of PN participants’ role is successfully connecting MSM participants with the right people and services to help them solve their challenges.

“We haven’t really done anything about the fact that I have no permanent job. But most of my challenges about everyday life, like food, like checking on me, he has helped me with solutions about those. He helps me with the rest of my challenges.”

(Participant 7)

PN participants accomplished this through consistently checking-in with MSM participants and asking about their challenges: “*I must call the participant every day to know about the participant’s situation*, *and we meet every Sunday in our support group*” (PN 1). This also occurred through linking MSM participants with relevant health workers and services: “*Sometimes I bring [a psychologist] to our meetings and she talks to my [participants] and colleagues*” (PN 3). As a result of this support, MSM participants expressed feeling empowered and being given the skills needed to engage with health workers in the future.

“He [the PN] does empower me. He helps me to be in contact with other medical personnel, and he really advises me on what to do, especially when he engages with them on my behalf to make sure that I can adhere to my treatment.”

(Participant 11)

According to both PN and MSM participants, the PN pilot program was integral in helping participants obtain essential material resources like food, transportation, and housing assistance.

“For example, when I have a challenge and I call him [the PN], he helps me. I can call when I have no food, or when I have a particular problem and he helps me out.”

(Participant 12)

As previously mentioned, MSM participants experienced a variety of challenges to ARV adherence. When assisting participants with ARV adherence, some PN participants believed it was crucial to provide extra emotional support to MSM participants beginning ARVs for the first time. They did this because they themselves knew firsthand from their own experiences how difficult beginning ARVs can be.

“I bring them to stay with me during their first weeks on medication. It is because I don’t want them to stop taking the medication from the very beginning. This is because I went through that first period, and it is a tough period. So, I need to give them extra care.”

(PN 1)

## Discussion

This study sought to explore the potential care benefits of a pilot program using patient navigators (PNs) to assist men who have sex with men (MSM) recently diagnosed with HIV in Kampala, Uganda. From the multiple ways that PN participants ensured that MSM participants attended medical appointments, adhered to their ARV treatment protocol, and received help for their general medical needs, our findings demonstrate that in a Ugandan context, PN participants were helpful in not only getting MSM participants to achieve the HIV Care Continuum stage of retention in care, but also effectively assisted MSM participants in reaching the other Care Continuum stages of diagnosis, linking participants to care, and receiving HIV medical care [1]. Although it is not known how many participants reached viral suppression while enrolled in the PN pilot program, PN participants illustrated via activities such as counselling and ARV adherence reminders that viral suppression was something that they were helping MSM participants to work towards. The emerging theme *Cultivating resilience and hope against enduring trauma and stress* highlights the barriers faced by HIV positive MSM and the important role played by the PN participants by giving MSM participants the tools needed to overcome future challenges they face as HIV positive MSM. Our findings also suggest that PN participants need psychosocial support to cope with participant expectations and demands.

### Experiencing a variety of barriers

In this study, MSM participants experienced a range of barriers to care, including HIV and LGBT related stigma, lacking money for necessities, difficulty finding sustainable work, struggling to take ARVs, fearing ARV side effects, and experiencing ARV side effects. These barriers were rooted in social, resource, or emotional challenges and had the potential of causing MSM participants to drop off the HIV Care Continuum. Except for LGBT related stigma, these barriers were not unique to MSM and have been similarly seen in other studies about the experiences of people living with HIV (PLHIV) in Uganda [17–20, 27].

As an intervention, PNs represent a type of peer support [28–30]. Previous studies on the benefits of peer support for PLHIV in the United States, United Kingdom, Uganda, Nigeria, Mozambique, Kenya, and South Africa found that peer support—when provided in conjunction with consistent medical care—yielded high rates of retention in care, when compared to solely providing in-clinic follow ups [28–30]. Our results support previous findings that PNs are effective in bridging gaps between different entities within a health system, connecting individuals to health services, improving health literacy, and reducing patient fears [6, 8, 9]. These study results are similar to previous research findings in that the PN pilot both engaged MSM participants within every facet of the Continuum of Care and offered protective factors against potential challenges to medication adherence through providing a social support network [8, 31]. In Uganda, among heterosexually identified PLHIV, it has been previously observed that these individuals both created and linked networks that consisted of family, friends, other PLHIV, religious leaders, neighbors, and health workers [31]. The social capital from these naturally occurring networks was found to provide protection factors against risks factors for ARV non-adherence by helping with food assistance, transportation costs, information about HIV, stigma management, status disclosure, medication shortages, and ARV adherence [31]. Our results build on this finding because societal stigma against LGBT individuals and the illegality of same-sex sexual practices prevent MSM from accessing these networks. MSM participants within our study were wary of others knowing their HIV status because they viewed it as inviting questions about how they acquired HIV and who their sexual partners were. Our findings suggest that PN participants via the PN pilot program created formal networks for MSM participants that proxied the informal familial-communal networks used by heterosexual PLHIV. Within these formalized networks, MSM participants were assisted with their social, emotional, and resource needs through attending MSM friendly clinics, visiting LGBT organizations, and engaging with familial-like networks that consisted of PN participants and other HIV positive MSM.

### Receiving the tools to overcome future challenges

Through PN participants, the health professionals they engaged with, and counselling sessions they participated in, MSM participants received information about how to live well with HIV. From participating in the PN pilot, MSM participants obtained skills like how to take their ARVs, how to refill their prescriptions, how to interact with LGBT sensitized health professionals, how to navigate the health system, and how to advocate for themselves within healthcare settings. Similar findings have been found in other PN interventions [4–9]. Additionally, MSM participants in the PN pilot program shared their newly gained information and skills with other MSM in their circles. These findings shed light on how PN programs could benefit other MSM communities in high discrimination settings. It is important to note that in these settings, it has been documented that HIV prevention and control efforts are often hindered due to inaccuracies and scarcity of MSM and HIV positive population data and difficulties reaching MSM individuals [5, 13, 32]. Based on our findings, it could be suggested that PN interventions in these settings are well suited to overcome these two challenges as they can use both HIV positive and negative MSM to provide information and services to other individuals in their community.

### PNs needing psychosocial support to cope patient demands

When interviewed, PN participants expressed needing psychosocial support to offset both the high level of expectations and demands placed on them by MSM participants, to process their traumas and challenges, and to manage negative emotions that arose when they were unable to meet participant needs. For some PN participants, hearing about HIV or sexuality related participant traumas and challenges caused them to relive similar experiences that they themselves had gone through previously. These findings illustrate the potential needs of PN participants working in high-stigma contexts where participants have limited access to social, economic, and emotional support. These findings corroborate previous observations by Li et al. [33] who found that among HIV positive HIV service providers in Canada, many individuals reported needing more psychosocial support services after encountering challenges related to the transition from HIV service recipient to HIV service provider [33]. Some of these challenges included losing access to support groups when support group members became their clients, losing access to counsellors who had now become their colleagues, and having limited options in peers they could confide in as a result of being part of a small community [33]. Moreover in similarity to our study, Li et al. [33] also found that service providers adapted to these challenges by confiding in colleagues who were also peers and service providers, or by confiding in mental health professionals if they had access to them [33]. It is important to note that even though not all the PN participants in our study were HIV positive, the findings of Li et al. [33] are applicable because PN participants in our study provided services for members of a marginalized community that they themselves were part of.

### Sustainability of PN programs

The PN pilot program discussed within this study took place from January 2019 to December 2020, where MSM participants received a variety of material, emotional, and social support from PN participants with the goal of helping them remain within the HIV Care Continuum. Although this pilot was successful in meeting this goal, further study is needed on the long-term sustainability and efficacy of PN interventions. This suggestion is similarly highlighted in a review of seven PN programs by Roland et al. [10], who found that participants were apprehensive about leaving their respective programs because they wanted to continue their relationship with their PNs, and because they still needed long term assistance with nonmedical support [10, 34, 35].

Additionally, we recommend further study on the long-term financial feasibility of PN programs. As previously mentioned, PN participants within our study were able to help MSM participants remain within the HIV Continuum of Care by providing material resources, emotional support, and social support at an individual level. Because this individualized approach to support was key to this pilot’s success, further research is recommended to understand the financial feasibility of this individualized approach for long term and larger scale programs. This recommendation was similarly made by Shade et al. [36], who in their review of 16 PN interventions across the United States found that among these interventions (all of them taking place over 11–12 months) most of their intervention costs went towards providing tailored support to patients at the individual level [36].

### Methodological considerations

A few important limitations exist within this study. First, even though Malterud’s concept of information power was used to decide when study recruitment finished, because this study contains the perspective of only four PN participants it is not possible to know if including additional PNs within the pilot program and research study would have further strengthened this study’s design [22]. Second, since June 2019 and November 2019 interviews were analyzed together, it was not possible to establish temporality and pinpoint exactly when participants moved to different stages within HIV Care Continuum, when participants became adjusted to life with HIV, and when in the HIV Care Continuum participants experienced challenges and setbacks. Moreover, since this study was set in Kampala, it is possible that study results might not be applicable to rural settings in Uganda where contextual differences may be present (e.g., less resources being available to MSM or PLHIV, and a lower population density of MSM or PLHIV).

This study also has several strengths. The analytical process allowed for multiple research team members to examine the analytical choices made, thus increasing the confirmability of study results. Moreover, the use of interpreters allowed interviewees to both clearly understand the questions posed to them and describe their experiences as accurately as possible. Lastly, research team members’ previous experience and knowledge regarding the topics of HIV, key populations, Uganda, and sexual and reproductive health and rights enabled a nuanced and informed approach to this study’s design, execution, and analysis [22].

## Conclusion

In Kampala, Uganda, men who have sex with men (MSM) living with HIV experience a variety of material, emotional, and social challenges that place them at risk for falling off the HIV Care Continuum. MSM living with HIV benefitted from patient navigators (PNs) via a PN pilot program by being provided with the support, skills, and resources needed to overcome these barriers. Among participants, the assistance from the PNs helped to cultivate the resilience needed to remain within the HIV Care Continuum. While participating in the PN pilot program, both PN and MSM participants reported positive experiences and occasional challenges. For PN participants, positive experiences included fulfillment from helping participants overcome obstacles and challenges, but they also reported feeling overwhelmed by the high number of MSM participant demands. For MSM participants, positive experiences included finding community among other HIV positive MSM but also challenges such as difficulty with ARV adherence. Based on our study results, further research is required on the psychosocial support needs of PNs and how the support needs of MSM participants change throughout their lifetime within the HIV Care Continuum. Additionally, study results also suggest that more research is needed on how the potential care benefits of using PNs might differ in rural contexts within Uganda.

## Supporting information

S1 Text MSM participant interview guide. (DOCX)

S2 Text PN participant interview guide. (DOCX)

## References

[1] US Department of Health & Human Services. What Is the HIV Care Continuum? 2021 [cited 2021 August 24]. https://www.hiv.gov/federal-response/policies-issues/hiv-aids-care-continuum

[2] BenBella D, Ghosh D. Combining Geospatial Analysis with HIV Care Continuum to Identify Differential HIV/AIDS Treatment Indicators in Uganda. Professional Geographer. 2021;73(2):213–29. doi: 10.1080/00330124.2020.1844573

[3] Windsor LC, Pinto RM, Lee CA. Interprofessional collaboration associated with frequency of life-saving links to HIV continuum of care services in the urban environment of Newark, New Jersey. BMC Health Services Research. 2020;20(1):1–9. doi: 10.1186/s12913-020-05866-3 33160344 PMC7648428

[4] Graham SM, Micheni M, Kombo B, Van Der Elst EM, Mugo PM, Kivaya E, et al. Development and pilot testing of an intervention to promote care engagement and adherence among HIV-positive Kenyan MSM. AIDS (02699370). 2015;29:S241–S9. doi: 10.1097/QAD.0000000000000897 26562813 PMC4706368

[5] Loya-Montiel MI, Davis DA, Aguilar-Martínez JM, Paz Bailey OA, Morales-Miranda S, Alvis-Estrada JP, et al. Making the Link: A Pilot Health Navigation Intervention to Improve Timely Linkage to Care for Men Who have Sex with Men and Transgender Women Recently Diagnosed with HIV in Guatemala City. AIDS & Behavior. 2019;23(4):900–7. doi: 10.1007/s10461-018-2328-6 30377979 PMC6691504

[6] Irma NR, Kristie NR, Kenneth SR. Driving the precision medicine highway: community health workers and patient navigators. Journal of Translational Medicine. 2019;17(1):1–4. doi: 10.1186/s12967-019-1826-2 30876478 PMC6419796

[7] Freund KM. Implementation of evidence-based patient navigation programs. Acta Oncologica. 2017;56(2):123–7. doi: 10.1080/0284186X.2016.1266078 28033027

[8] Janet JM, Kimberly AK, Mi-Suk Kang D, Alison OJ, Jacqueline C-Q, Alissa R. Patient navigators effectively support HIV-infected individuals returning to the community from jail settings. International Journal of Prisoner Health. 2017;13(3/4):213–8. doi: 10.1108/IJPH-08-2016-0037 28914126

[9] Pearl A, Lewis V, Brown T, Russell G. Patient navigators facilitating access to primary care: a scoping review. BMJ OPEN. 2018;8(3):e019252. doi: 10.1136/bmjopen-2017-019252 29550777 PMC5875656

[10] Roland KB, Higa DH, Leighton CA, Mizuno Y, DeLuca JB, Koenig LJ. Client Perspectives and Experiences With HIV Patient Navigation in the United States: A Qualitative Meta-Synthesis. Health Promotion Practice. 2020;21(1):25–36-. doi: 10.1177/1524839919875727 31597497 PMC6917848

[11] McBrien KA, Ivers N, Barnieh L, Bailey JJ, Lorenzetti DL, Nicholas D, et al. Patient navigators for people with chronic disease: A systematic review. PLoS ONE. 2018;13(2):1–33. doi: 10.1371/journal.pone.0191980 29462179 PMC5819768

[12] Van Walleghem N, MacDonald CA, Dean HJ. The Maestro Project: A Patient Navigator for the Transition of Care for Youth With Type 1 Diabetes. Diabetes Spectrum. 2011;24(1):9–13. doi: 10.2337/diaspect.24.1.9

[13] Kuhns LM, Johnson AK, Adetunji A, Kuti KM, Garofalo R, Omigbodun O, et al. Adaptation of evidence-based approaches to promote HIV testing and treatment engagement among high-risk Nigerian youth. PLoS ONE. 2021;16(10):1–18. doi: 10.1371/journal.pone.0258190 34614028 PMC8494297

[14] UNAIDS. Uganda Country Factsheet 2020 [cited 2021 August 24, 2021]. https://www.unaids.org/en/regionscountries/countries/uganda

[15] Doshi RH, Apodaca K, Ogwal M, Bain R, Amene E, Kiyingi H, et al. Estimating the Size of Key Populations in Kampala, Uganda: 3-Source Capture-Recapture Study. Journal of Medical Internet Research. 2019;21(8):N.PAG-N.PAG. doi: 10.2196/12118 31407673 PMC6771531

[16] Wolfgang H, Joseph B, John MS, Alex O, Jordan WT, Avi H, et al. HIV infection among men who have sex with men in Kampala, Uganda—a respondent driven sampling survey. PLoS ONE. 2012;7(5):e38143–e. doi: 10.1371/journal.pone.0038143 22693590 PMC3364961

[17] King R, Sebyala Z, Ogwal M, Aluzimbi G, Apondi R, Reynolds S, et al. How men who have sex with men experience HIV health services in Kampala, Uganda. BMJ Global Health. 2020;5(4). doi: 10.1136/bmjgh-2019-001901

[18] Kajubi P, Kamya MR, Raymond HF, Chen S, Rutherford GW, Mandel JS, et al. Gay and bisexual men in Kampala, Uganda. AIDS & Behavior. 2008;12(3):492–504. doi: 10.1007/s10461-007-9323-7 17968647

[19] Wanyenze RK, Musinguzi G, Matovu JKB, Kiguli J, Nuwaha F, Mujisha G, et al. “If You Tell People That You Had Sex with a Fellow Man, It Is Hard to Be Helped and Treated”: Barriers and Opportunities for Increasing Access to HIV Services among Men Who Have Sex with Men in Uganda. PLoS ONE. 2016;11(1):1–19. doi: 10.1371/journal.pone.0147714 26808653 PMC4726486

[20] King R, Barker J, Nakayiwa S, Katuntu D, Lubwama G, Bagenda D, et al. Men at Risk; a Qualitative Study on HIV Risk, Gender Identity and Violence among Men Who Have Sex with Men Who Report High Risk Behavior in Kampala, Uganda. PLoS ONE. 2013;8(12):1–8. doi: 10.1371/journal.pone.0082937 24358239 PMC3866199

[21] Raymond HF, Kajubi P, Kamya MR, Rutherford GW, Mandel JS, McFarland W. Correlates of unprotected receptive anal intercourse among gay and bisexual men: Kampala, Uganda. AIDS & Behavior. 2009;13(4):677–81. doi: 10.1007/s10461-009-9557-7 19495955 PMC2829255

[22] Malterud K, Siersma VD, Guassora AD. Sample Size in Qualitative Interview Studies: Guided by Information Power. QUALITATIVE HEALTH RESEARCH. 2016;26:1753–60. doi: 10.1177/1049732315617444 26613970

[23] Graneheim UH, Lindgren B-M, Lundman B. Methodological challenges in qualitative content analysis: A discussion paper. Nurse Education Today. 2017;56:29–34. doi: 10.1016/j.nedt.2017.06.002 28651100

[24] Graneheim UH, Lundman B. Qualitative content analysis in nursing research: Concepts, procedures and measures to achieve trustworthiness. Nurse Education Today. 2004;24(2):105–12. doi: 10.1016/j.nedt.2003.10.001 14769454

[25] O’Brien BC, Harris IB, Beckman TJ, Reed DA, Cook DA. Standards for Reporting Qualitative Research: A Synthesis of Recommendations. Academic Medicine. 2014;89(9):1245–51. doi: 10.1097/ACM.0000000000000388 24979285

[26] World Medical Association. WMA Declaration of Helsinki- Ethical Principles for Medical Research Involving Human Subjects 2013 [updated September 6, 2022; cited 2021 February 5]. https://www.wma.net/policies-post/wma-declaration-of-helsinki-ethical-principles-for-medical-research-involving-human-subjects/

[27] Musinguzi G, Bastiaens H, Matovu JKB, Nuwaha F, Mujisha G, Kiguli J, et al. Barriers to Condom Use among High Risk Men Who Have Sex with Men in Uganda: A Qualitative Study. PLoS ONE. 2015;10(7):1–13. doi: 10.1371/journal.pone.0132297 26172374 PMC4501754

[28] Nyoni T, Sallah YH, Okumu M, Byansi W, Lipsey K, Small E. The effectiveness of treatment supporter interventions in antiretroviral treatment adherence in sub-Saharan Africa: a systematic review and meta-Analysis. AIDS Care. 2020;32:214–27. doi: 10.1080/09540121.2020.1742870 32196385

[29] Brough G. Peer support in HIV care. HIV Nursing. 2016;16(2):26–8.

[30] Berg RC, Page S, Øgård-Repål A. The effectiveness of peer-support for people living with HIV: A systematic review and meta-analysis. PLoS ONE. 2021;16(6):1–24. doi: 10.1371/journal.pone.0252623 34138897 PMC8211296

[31] Nanfuka EK, Kyaddondo D, Ssali SN, Asingwire N. Social capital and resilience among people living on antiretroviral therapy in resource-poor Uganda. PLoS ONE. 2018;13(6):1–21. doi: 10.1371/journal.pone.0197979 29889849 PMC5995438

[32] Hladik W, Sande E, Berry M, Ganafa S, Kiyingi H, Kusiima J, et al. Men Who Have Sex with Men in Kampala, Uganda: Results from a Bio-Behavioral Respondent Driven Sampling Survey. AIDS & Behavior. 2017;21(5):1478–90. doi: 10.1007/s10461-016-1535-2 27600752

[33] Li AT-W, Wales J, Wong JP-H, Owino M, Perreault Y, Miao A, et al. Changing access to mental health care and social support when people living with HIV/AIDS become service providers. AIDS Care. 2015;27(2):176–81. doi: 10.1080/09540121.2014.940269 25069033

[34] Fuller SM, Koester KA, Maiorana A, Steward WT, Broaddus MR, Lass K, et al. "I don’t have to do this all by myself": Systems Navigation to Ensure Continuity of HIV Care for Persons Leaving Prison. 2019. 10.1007/s10461-018-2050-4 29442194

[35] Broaddus MR, Hanna CR, Schumann C, Meier A. "she makes me feel that I’m not alone": Linkage to Care Specialists provide social support to people living with HIV. AIDS Care—Psychological and Socio-Medical Aspects of AIDS/HIV. 2015;27(9):1104-7-7. doi: 10.1080/09540121.2015.1028882 25854534 PMC4596723

[36] Shade SB, Kirby VB, Stephens S, Moran L, Charlebois ED, Xavier J, et al. Outcomes and costs of publicly funded patient navigation interventions to enhance HIV care continuum outcomes in the United States: A before-and-after study. 2021. 10.1371/journal.pmed.1003418 PMC8118317 33983925

