# Supplementary material for: Cultivating resilience and hope: A qualitative study of a pilot program using patient navigators to assist men who have sex with men with retention in the HIV care continuum in Uganda
Source: PLOS Glob Public Health. 2023 Jan 19;3(1):e0001475. doi: 10.1371/journal.pgph.0001475 (PMC10021195; doi:10.1371/journal.pgph.0001475)
Supplement: S1 Text — (DOCX) [file pgph.0001475.s001.docx]

***S1. Interview guide for MSM participants***

*The research questions that have guided the development of this interview guide are as follow:*

*1) What are the experiences of MSM participants with HIV in patient navigation programs?*

*2) What is the impact of patient navigators on the challenges MSM participants encounter in the HIV care continuum?*

**Opening question:**

1. Would you like to tell me about yourself?

Probes:

- - Age
  - Family/relationship situation
  - Self-identified sexual orientation
  - Educational/professional background

**Information about their experience living with HIV**

1. Thinking back to when you were diagnosed with HIV, what were some of the conversations you had with health care providers, family or friends?

Probes:

- What did you talk about?
- Where was this (special HIV hospital, general hospital, take home test)?
- When was this?
- How was the experience?
  - How did the staff treat you?
  - Did you receive the support and help you needed?

1. What challenges do you face managing your health?

Probes:

- Think of a day this past month that was particularly distressing for you, what about that day was challenging?

1. Are you currently on any HIV medication?

- If yes: For how long have you been on this medication?
- If no, why not?

1. If you think back about your experience with the HIV testing and care system, before participating in this program, what has it been like?

Probes:

- Describe a time when you were in contact with the HIV testing and care system
- Have you been comfortable explaining your sexual practices and related illnesses to health workers?

**Their individual relationship with their patient navigator**

1. How would you describe your relationship with your patient navigator?

Probes:

- - Are you close? Do you feel that you can trust him? Do you feel that he can represent your interests? If yes, how? If no, why?
  - How often do you meet?

1. Try to recall a time when the patient navigator did something particularly helpful. Please tell me about that time.

Probes:

- - What did the patient navigator do, specifically? How was it helpful?

**Their experience within the patient navigator pilot program**

1. Why did you choose to participate in this program?

Probes:

- Was there a specific way that you felt a patient navigator could help you?

1. We discussed the challenges that you face related to manging your health, does the patient navigator make a difference with regard to those challenges?

- If yes, how?
- If no, why?

1. How does being a participant in the PN program affect you?

Probes:

- Did working with the patient navigator help you get HIV care?
- Did the patient navigator influence how you feel about living with HIV? If yes, how? If no, why do you think so?

1. What effect does the patient navigator have on your interactions with health care professionals (nurse, doctors, pharmacists, social workers etc.)?
2. Does the patient navigator empower you to manage your health?

- If yes, how?

Probes:

- Information about your disease
- Help to understand the health system and your patient rights
- Help to interact with health care providers
- If no, why do you feel this way?

**Closure**

1. Is there anything else you would like to tell me about your experience?
2. What advice would you give to another self-identified MSM who has been diagnosed with HIV?
3. Do you have any questions or concerns at this time?
