# Supplementary material for: Cultivating resilience and hope: A qualitative study of a pilot program using patient navigators to assist men who have sex with men with retention in the HIV care continuum in Uganda
Source: PLOS Glob Public Health. 2023 Jan 19;3(1):e0001475. doi: 10.1371/journal.pgph.0001475 (PMC10021195; doi:10.1371/journal.pgph.0001475)
Supplement: S2 Text — (DOCX) [file pgph.0001475.s002.docx]

***S2. Interview guide for patient navigators***

*The research questions that have guided the development of this interview guide are as follow:*

*1) What are the experiences of MSM participants with HIV in patient navigation programs?*

*2) What is the impact of patient navigators on the challenges MSM participants encounter in the HIV care continuum?*

**Opening question:**

1. Would you like to tell me about yourself?

Probes:

- - Age
  - Family/relationship situation
  - Self-identified sexual orientation
  - Educational/professional background

**Their motivation and ideals**

As you know, this research study is about patient navigators and their role in assisting men who have sex with men in the first stage after being diagnosed with HIV.

1. Could you share with me why you chose to become a patient navigator?

Probes:

- Specific incident in your life that affected decision

1. How would you describe your role as a patient navigator?

Probes:

- Duties and responsibilities

1. What are the most important qualities that a patient navigator should have to be successful?

- Why these?

**Their perceptions of pilot participants**

Now we will talk about the participants that you are working with. We are mainly interested in your experience as a patient navigator and we do not, at any point, request you to disclose information about the participants that would threaten their anonymity. Should you do so, we will remove this information from the interview.

1. How many participants do you work with?

Probes:

- How long have you worked with these XX participants?
- Were you assigned all participants are the same time?

1. In your experience, what are the main challenges that these participants face in order to manage their HIV infection?

Probes:

- What kind of problems do they bring to you?
- How do they approach you?
- Do you believe there are other issues that they face but that they don’t bring to you? Why?

1. Do you feel that you in your role as patient navigator have an impact on the challenges that the participants face in order to manage their HIV infection?

- If yes, describe how you have an impact? What has been the result for the participant?
- If no, why do you feel so?

**Their relationship with pilot participants**

1. How would you describe your relationship with the participants you have been assigned?

Probes:

- Could you give an example of how you first established contact and trust with one of your participants?

1. How do you motivate your participants to continue HIV treatment and care?

Probes:

- Could you give me an example of a situation when this has happened?

1. As you reflect on working with these participants, I am sure there have been some high and low times. Can you focus on a high point?

Probes:

- How would you describe your relationship with the participant?

1. And a low point?

Probes:

- How would you describe your relationship with the participant?

**Their experiences within the patient navigator pilot program**

1. What elements of the program have worked well?

Probes:

Why do think this worked well?

1. What would you say have been the greatest challenges of fulfilling your role as patient navigator?

Probes*:*

- Think of a day this past month that was particularly distressing for you, what about that day was challenging?

1. What kind of support do you receive for providing these services?

**Closure**

1. Is there anything else you would like to tell me about your experience?
2. What advice would you give to other programs that try to address HIV care retention among MSM?
3. Do you have any questions or concerns at this time?
